# Curcumin as a Multifunctional Spice Ingredient against Mental Disorders in Humans: Current Clinical Studies and Bioavailability Concerns

**DOI:** 10.3390/life14040479

**Published:** 2024-04-05

**Authors:** Maria Spanoudaki, Sousana K. Papadopoulou, Georgios Antasouras, Konstantinos A. Papadopoulos, Evmorfia Psara, Theofanis Vorvolakos, Evangelos Solovos, Maria Chrysafi, Michalis Psallas, Maria Mentzelou, Despoina Ourda, Constantinos Giaginis

**Affiliations:** 1 Department of Nutritional Sciences and Dietetics, School of Health Sciences, International Hellenic University, 57400 Thessaloniki, Greecesouzpapa@gmail.com (S.K.P.); 2 424 General Military Hospital of Thessaloniki, 54621 Thessaloniki, Greece; konpapadopoulos@yahoo.com (K.A.P.); esolovos@hotmail.com (E.S.); mikedoc70@yahoo.gr (M.P.); 3 Department of Food Science and Nutrition, School of Environment, University of Aegean, 81400 Lemnos, Greece; g.antasouras@gmail.com (G.A.); fnsd21013@fns.aegean.gr (E.P.); fnsm22030@fns.aegean.gr (M.C.); maria.mentzelou@hotmail.com (M.M.); 4 Department of Psychiatry, School of Medicine, Democritus University of Thrace, 68100 Alexandroupolis, Greece; tvorvola@med.duth.gr; 5 Department of Physical Education and Sport Science, Aristotle University of Thessaloniki, 54124 Thessaloniki, Greece; despoino@phed.auth.gr

**Keywords:** curcumin, mental disorders, depression, anxiety, stress, bioavailability, nanoformulation, mood disorders, solubility, piperine

## Abstract

Background: Mental disorders in terms of depression, anxiety, and stress are one of the major causes of burden globally. Over the last two decades, the use of plant-based substances in the treatment of mental disorders in combination or not with medication has increasingly attracted the interest of the scientific research community. However, even if there is a plethora of naturally occurring bioactive compounds, most of them have low bioavailability, rendering them unable to insert into the bloodstream to exert their biological activities. Methods: This is a comprehensive narrative review that critically summarizes and scrutinizes the new approaches to the treatment of mental disorders using curcumin, also highlighting its bioavailability properties. The most accurate were searched using effective and relevant keywords. Results: This narrative review reveals substantial evidence that curcumin can exert significant effects on several mental disorders. However, despite the low cost, the extensive and confirmed potency of curcumin and its involvement in signaling pathways and the scientifically confirmed data regarding its molecular mechanisms of action against mental disorders, this naturally occurring compound presents low oral bioavailability. Pharmaceutical technology has provided solutions to increase the bioavailability of curcumin. Combination with piperine, galactomannosides, liposomal formulation or nanoformulation overcomes the bioavailability and solubility disadvantages. Conclusions: Although curcumin demonstrates anti-anxiety, anti-depressive and anti-stress properties, studies on humans are limited and heterogeneous. Further research is highly recommended to determine the most functional formula, dose, duration, and possible side effects of curcumin on mental disorders in humans. Based on the current knowledge, the curcumin nanoformulation and Theracurmin, a form of colloidal submicroscopic particles, seem to be the most effective bioavailable formulations, which may be examined in future clinical human studies.

## 1. Introduction

Mental disorders are one of the main causes of burden worldwide. The global number of disability-adjusted life-years (DALYs) because of mental disorders increased from 80.8 million (95% uncertainty interval (UI) 59.5–105.9) to 125.3 million (93.0–163.2), and the proportion of disability-adjusted life-years in the world assigned to mental disorders raised from 3.1% to 4.9% [1]. Over the last two decades, the use of plant-based substances in the treatment of mental disorders in combination or not with medication has attracted the interest of international scientific research. Curcumin is a non-flavonoid polyphenolic compound derived from the root of the plant *Curcuma longa* (turmeric), which is commonly used in Indian and Chinese folk medicine for the treatment of rheumatism, inflammation, asthma, cancer, and wounds [2]. Curcumin’s pharmacological properties include interactions with several intracellular signaling pathways and also control mood profiles. Additionally, curcumin can exert antioxidant, neurotrophic and anti-inflammatory actions, regulating health issues related to neurodegeneration [3]. 

The potential targets of curcumin are based on data from a few studies on mental disorders in humans and mainly include some proteins implicated in the pathophysiology of depression, anxiety, stress (social, occupational and especially post-traumatic stress), bipolar disorder and schizophrenia. These targets have focused on transcription factors triggered by stressors and protein kinases (e.g., PKC, PKA), pro-inflammatory cytokines, growth factors, enzymes, inflammatory mediators and anti-apoptotic proteins [4]. Notably, curcumin has been found to regulate neurotransmitters in the brain, especially serotonin, dopamine, and norepinephrine, exerting a critical impact on mood regulation by increasing their levels and enhancing its antidepressant effect [5]. It also exhibits potent anti-inflammatory properties, taking part in various inflammatory pathways, including cytokine modulation and reduction in inflammatory markers [6,7]. By reducing neuro-inflammation, curcumin may alleviate depressive symptoms. Moreover, chronic inflammation has been linked to psychological distress not only concerning anxiety and depression but also neurosis such as schizophrenia [8].

By promoting neurogenesis—the growth of new neurons—and enhancing neuroplasticity in animal models, curcumin can potentially reverse the neuronal damage associated with depression, enhancing the brain’s ability to adapt to stress and other environmental factors [9]. It should be noted that oxidative stress plays a role in the pathophysiology of depression, anxiety, bipolar disorder and schizophrenia. In this aspect, curcumin acts as an antioxidant by scavenging free radicals, inhibiting the production of reactive oxygen species (ROS), and reducing oxidative stress, thus protecting brain cells from damage and contributing to its neuroprotective effects [10]. The hypothalamic–pituitary–adrenal (HPA) axis is involved in the body’s response to stress. Dysregulation of this system is associated with depression. In this context, curcumin has been shown to regulate the HPA axis, potentially reducing the impact of stress on the body and brain [10] by modulating cortisol levels and promoting a potential effect on stress response [11].

In addition, curcumin interacts with several signaling pathways involved in mood regulation, such as the cyclic adenosine monophosphate (cAMP), response element-binding protein (CREB) pathway and the brain-derived neurotrophic factor (BDNF) pathway [12]. These interactions may contribute to its antidepressant effects by regulating the expression of genes related to mental and neuronal health [9]. Emerging research has shown that gut microbiota plays a crucial role in mental health. Notably, curcumin may positively affect the gut–brain axis by modulating gut microbiota, which may influence brain function and potentially alleviate depressive symptoms [13].

Although curcumin has several promising beneficial effects on the treatment of mental disorders, the international literature has focused on its low bioavailability and adsorption when given orally in humans [14]. The low oral bioavailability of curcumin may be due to its low intestinal absorption, high metabolic rate and rapid elimination because most of the substance is eliminated from the human body without being metabolized [15]. In the last few years, the increasing progress in pharmaceutical biotechnology has highlighted the efficiency of curcumin’s chemical formulation to face bioavailability and absorption problems in order to manage chronic diseases more effectively [16]. 

As far as we know, the scientific field on curcumin’s effect on mental disorders in humans is rather scarce as most of the studies have been conducted in animals. In this aspect, the aim of our review was to critically summarize and scrutinize the effectiveness of curcumin supplementation on mental disorders in humans including depression, bipolar disorder, anxiety, stress, overcoming absorption and bioavailability disadvantages.

## 2. Materials and Methods

Comprehensive research was conducted on PubMed, Embase, Scopus, Cochrane Library (Clinical Trials) and Web of Science in terms of “curcumin and mental disorders”, “curcumin and anxiety”, “curcumin and stress disorder”, “curcumin and depression or/and major depression disorder or/and bipolar disorders” in humans or individuals and “curcumin bioavailability and/or metabolism and/or chemical properties”. Exclusion criteria were studies published over the last decade, studies including animals, not written in the English language, reviews, systematic reviews and grey literature. The retrieved studies were also carefully checked for related studies quoted in their text. Articles on curcumin were not included if they did not meet the main theoretical content of our study.

**Figure 1:**
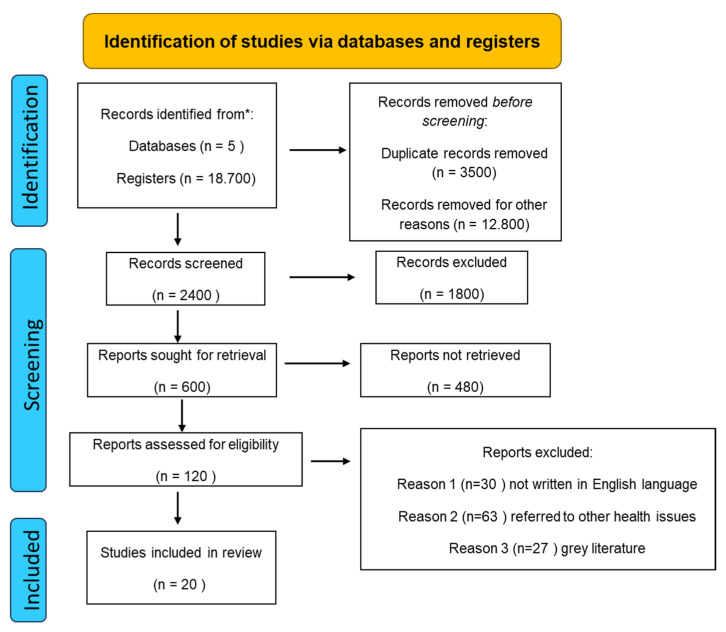
Flow chart diagram: searching strategy. * PubMed, Embase, Scopus, Cochrane Library (Clinical Trials) and Web of Science.

All authors acted as reviewers. Thus, in order to increase consistency, all authors, working in pairs, reviewed all retrieved publications, discussed their findings and adjusted the data extraction manually before starting the screening for this review. They alternately assessed the titles, abstracts and then the full texts of all publications retrieved from their searches for potentially relevant publications with good methodology and credible study design. A data charting form was developed jointly by two reviewers (M.S. and C.G.), who independently recorded the data and discussed the findings and the charting format in an iterative process. In Figure 1, a flow chart diagram is presented illustrating the selection of studies.

## 3. Curcumin’s Chemical Properties

Curcumin is a natural non-flavonoid polyphenolic compound found in the rhizomes of turmeric. It is known for its bright yellow color and has a wide range of chemical properties that contribute to its biological activities [17]. Curcumin has a chemical formula of C_21_H_2_0O_6_ and consists of two aromatic (phenolic) rings linked by a chain of seven carbon atoms. Its polyphenolic structure, characterized by phenolic rings and functional groups, contributes to its antioxidant properties [17]. More to the point, curcumin contains two phenolic hydroxyl (-OH) groups attached to the phenyl rings. These hydroxyl groups contribute to its antioxidant activity by scavenging free radicals and combating oxidative stress [18]. Two methoxy (-OCH3) groups are also present in its phenyl rings. These groups contribute to curcumin’s stability and lipophilic nature, affecting its solubility and bioavailability [8].

Furthermore, curcumin belongs to the class of compounds known as curcuminoids. Its hydrophobic and pH-dependent nature is responsible for its limited bioavailability when taken orally. However, it is soluble in organic solvents such as ethanol and dimethyl sulfoxide (DMSO). Alkaline conditions promote its solubility, affecting its absorption and stability [19]. Curcumin also exists in multiple forms or isomers, with the most common being curcumin, dimethoxy–curcumin and bis–dimethoxy–curcumin. These isomers have slightly different chemical properties and biological activities and coexist in turmeric extracts [20]. While curcumin is the most researched and commonly known isomer, both dimethoxy–curcumin and bis–dimethoxy–curcumin also contribute to the overall biological effects of turmeric and its derived products [21]. The presence of these isomers may influence the overall health benefits and bioactivities attributed to turmeric or curcumin supplements. Curcumin can also be met in two tautomeric forms: β-diketone and β-keto-Enol, which is more stable and more antioxidant active than β-diketone, as it has three chelating metal areas, promoting scavenging free radicals [22,23].

Curcumin’s ability to be absorbed and utilized by the body is a critical factor in determining its effectiveness as a therapeutic agent. It is poorly absorbed in the gastrointestinal tract, leading to low systemic bioavailability [24]. It undergoes rapid metabolism and elimination, limiting its concentration in the bloodstream. Once absorbed, curcumin undergoes extensive metabolism in the liver and intestinal wall, leading to the formation of various metabolites, many of which are conjugated and quickly excreted from the body [8]. Curcumin undergoes phase II metabolism, primarily glucuronidation and sulfation, leading to the formation of curcumin–glucuronide and curcumin sulfate, among other metabolites. These metabolites are more water soluble and easily excreted by the kidneys [25]. The rapid metabolism and elimination of curcumin contribute to its short half-life in the bloodstream, limiting at a significant level its systemic exposure and bioavailability [25,26].

**Figure 2:**
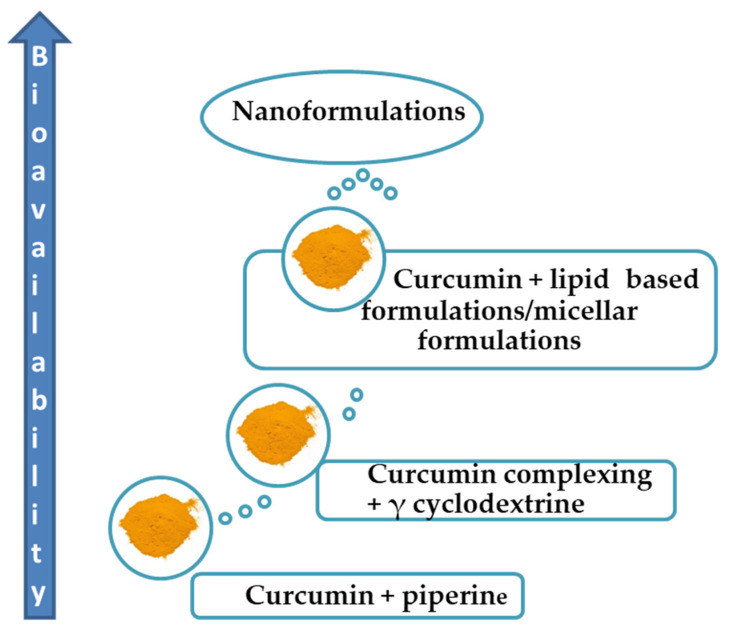
Potential forms of curcumin with improved oral bioavailability.

In view of the above considerations, enhancing curcumin’s bioavailability in humans is crucial to optimize its therapeutic efficacy. In Figure 2, the potential forms of curcumin with improved oral bioavailability are depicted. Several strategies and approaches have been explored to improve curcumin’s absorption and bioavailability. Co-administration of curcumin with piperine, a compound found in black pepper, has been shown to enhance curcumin’s bioavailability. In fact, piperine can inhibit certain enzymes responsible for curcumin metabolism, thereby increasing its concentration in the bloodstream [27]. Moreover, combining curcumin with fats, oils or lecithin can improve its solubility and absorption, enhancing its bioavailability. Notably, lipid-based formulations protect curcumin from degradation in the gastrointestinal tract, improving its absorption [28].

Complexing curcumin with cyclodextrins may also enhance its water solubility and stability, thereby improving its bioavailability. Substantial evidence has shown that γ-cyclodextrin-complexed curcumin formulations increase absorption and systemic exposure, contributing to higher bioavailability. Particularly, γ-cyclodextrin enhances the oral and metabolic bioavailability of curcumin and tetrahydro–curcumin, respectively. This is most likely ascribed to the improved uptake of cyclodextrin-conjugated curcumin, which has been confirmed by in vitro studies, its metabolic turnover and the prolonged half-life of tetrahydro–curcumin in plasma [29]. Micellar formulations can also provide solubility and absorption in the gastrointestinal tract, improving curcumin’s bioavailability and systemic exposure [30]. Nanotechnology-based formulations utilize nanoparticles to encapsulate curcumin, improving its stability and bioavailability. Nano-sized curcumin particles increase water solubility, protect against degradation and enhance absorption in the gastrointestinal tract, leading to improved systemic exposure [25].

## 4. Results

### 4.1. Depression

Depression is a complex and multifaceted mental health disorder that affects millions of people worldwide. It is characterized by persistent feelings of sadness, hopelessness and a loss of interest or pleasure in activities. The pathophysiological mechanisms governing depression disease are not fully understood yet, but it is believed to involve a combination of genetic, neurobiological, psychological and environmental factors [31,32]. Studies on brain Magnetic Resonance Imaging (MRI) and Computed Tomography (CT) have identified structural and functional differences in the brains of individuals suffering from depression. The hippocampus, which is crucial for mood regulation, often shows reduced volume in depressed individuals. The prefrontal cortex, which plays a critical role in decision making and emotional regulation, is also implicated [33]. Moreover, there is emerging evidence suggesting a link between inflammation and depression. In this aspect, chronic inflammation may affect the brain, leading to the development of depressive symptoms [34]. This has led to the expansion of the “cytokine hypothesis” and of acute proteins of depression. Interestingly, depressed patients have elevated levels of interleukine-6 (IL-6) and c-reactive protein (CRP), in serum, while IL-10 has negatively been correlated with depressive symptoms and disease progression [35].

Stress hormones such as cortisol also play a crucial role in mood regulation. In individuals with depression, the HPA axis seems to be dysregulated, leading to abnormal cortisol levels and stress responses [36]. Chronic inflammation and immune system dysfunction have also been associated with depression. It is assumed that inflammation may alter brain function and neurotransmitter systems, contributing to the development of depressive symptoms of diverse severity [34]. On the other hand, the pathophysiology of major depression involves intricate molecular mechanisms within the central nervous system (CNS) and peripheral systems [37].

Although the exact mechanisms are still a subject of ongoing research, several key molecular factors have currently been identified. The serotonin hypothesis posits that alterations in serotonin (5-HT) neurotransmission exert a critical impact on depression. Reduced serotonin levels are frequently observed in individuals with major depression. Antidepressant medications, particularly selective serotonin reuptake inhibitors (SSRIs), act by increasing serotonin availability within the synaptic cleft [38]. In addition, dysregulation of both norepinephrine and dopamine pathways in the brain also contributes to the development of depressive symptoms. Moreover, norepinephrine is associated with arousal and attention, while dopamine is linked to motivation and reward. Both these systems are targeted by various antidepressant medications [39].

A gradually increasing body of literature implicates neutrophins, a family of proteins that induce the survival, development and function of neurons, glutamate, aspartate receptors, cytokines and the immune system in neuroplasticity and synaptic function in major depression. BDNF is a neurotrophin that plays a crucial role in synaptic plasticity and neurogenesis. Reduced BDNF levels are associated with major depression [40]. This reduction may impair the brain’s ability to adapt and form new neural connections, which is essential for mood regulation [37]. Dysregulation of the glutamate system, particularly the glutamate and N-methyl-D-aspartate receptors, has also been implicated in depression. Excessive glutamate signaling and decreased neuroplasticity seem to contribute to the pathophysiology of the disease [41]. Glutamate is the major stimulating neurotransmitter in the brain, exerting an important effect in nearly all the core functions involved in depressive conditions. Postmortem investigations have highlighted data connecting glial cell aberrations, whose function is the synaptic removal of glutamate, and the background pathophysiology of mental disorders. Reduced glutamine/glutamate levels have also been detected in the cortex of depressed patients [42]. In addition, chronic stress and the HPA axis are strongly associated with depression. This can lead to increased levels of cortisol and alterations in the body’s stress response system [43].

### 4.2. Curcumin and Depression

**Figure 3:**
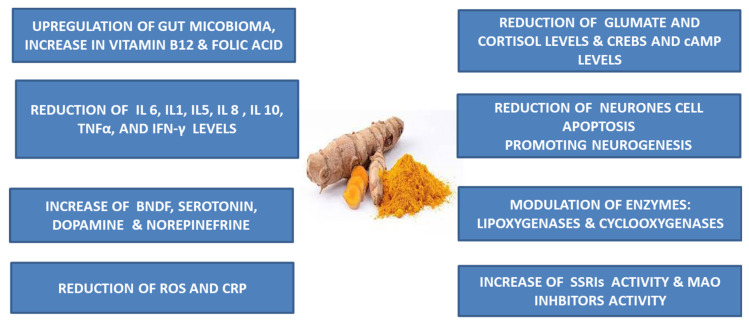
Potential molecular targets of curcumin to improve mental disorders symptoms and severity. Il: Interleukin, TNF-α: Tumor Necrosis Factor-α, INF: Interferon, ROS: Reactive Oxygen Species, CRP: C-Reactive Protein, BDNF: Brain-Derived Neurotrophic Factor, CREBS: cAMP Response Element Binding Proteins, cAMP: Cyclic Adenosine Monophosphate, SSRIs: Selective Serotonin Reuptake Inhibitors, MAO: Monoamine Oxidase.

Curcumin exhibits mechanisms in treating depression and anxiety by utilizing its anti-inflammatory, antioxidant and neuroprotective properties. It influences several molecular pathways involved in mood regulation and neuroplasticity, protecting the brain from oxidative stress and inflammation, which are often associated with these mental health conditions [44]. In Figure 3, the potential molecular targets of curcumin to improve mental disorders symptoms and severity are illustrated. Moreover, in Table 1, the currently available clinical studies evaluating the potential effects of curcumin against mental disorders are summarized.

Chronic inflammation is increasingly recognized as a contributing factor to depression. Curcumin has strong anti-inflammatory properties, suppressing the activity of inflammatory molecules like cytokines and enzymes involved in the inflammatory process [50]. By reducing inflammation, curcumin may alleviate the intensity of some symptoms associated with depression. Moreover, it can regulate peripheral biomarkers associated with depression and major depression progress, such as monoaminergic activity, cytokines circulation, stress hormones regulation and oxidative markers reduction [50].

Furthermore, supplementation with an increasing dosage rate from 500 mg to 1500 mg disorder, as indicated by significant improvements, occurs 12 and 16 weeks after the beginning of treatment [34]. Remarkably, curcumin has the ability to enhance anti-depressive medication effects like fluoxetine and bupropion in both animals and humans [54].

The chronic oral administration of 2 g of curcumin when combined with medication (escitalopram), provided a significant antidepressant behavioral response in depressed patients with a parallel reduction in the scores of the 17 items of the Hamilton Depression Rating Scale (HDRS) and the Montgomery–Asberg Depression Rating Scale (MADRS). Curcumin also reduced plasma levels of the inflammatory cytokines, such as tumor necrosis factor-α (TNF-α) and IL-1, elevated plasma levels of BDNF and decreased salivary cortisol levels in comparison to the placebo group [46]. Mild side effects (e.g., nausea) in the curcumin and escitalopram group were reported. Regarding monoamine-oxidase (MAO) action, this orange turmeric compound has demonstrated synergistic action with MAO inhibitors by increasing their activity [64]. However, a few studies in humans when 500 mg or 1000 mg per day of curcumin was supplemented orally demonstrated insignificant effects on depression even when combined with piperine and ellagic acid [49].

### 4.3. Bipolar Disorder

Bipolar disorder (BD) is a persistent, severe and prevalent mental illness characterized by alterations in behavior and mood such as irritability and depression [65]. Recurrent epidemiological studies have illustrated that BD has high frequencies of inflammatory comorbidities, including autoimmune disorders, chronic infections, cardiovascular diseases, and metabolic disorders [66]. Cytokine-based studies have established that BD is linked to chronic inflammation with a subsequent elevation of pro-inflammatory cytokine concentrations during episodes of mood alterations [67]. Increased levels of IL-6 and TNF-α have also been noted during manic episodes. IL-6 levels return to baseline levels after treatment with mood stabilizers, while TNF-α levels continue to remain high [68]. During manic episodes, IL-6, IL-5, interferon (INF)-γ and IL-8 were the upregulated pro-inflammatory cytokines, while only IL-6 was found to be raised during depression. In addition, BD is characterized by an imbalance between anti-inflammatory IL-10 and IL-6 [68].

Research has also identified some molecular mechanisms for explaining the bilateral relationship between BD and immune dysfunction. The most important mechanisms involve cytokine-induced monoamine alterations, augmented oxidative stress, pathological microglial hyperactivation, HPA axis hyperactivation, modifications in the microbiome-gut-brain axis, and sleep-related immune system alterations [69]. Moreover, impaired endocrine functions have extensively been studied in BD, including the analysis of hormone levels in blood and urine and the assessment of neuroendocrine systems, particularly the hypothalamic–pituitary–thyroid axis and the HPA axis [65]. In addition to brain disorders, BD has been associated with a variety of metabolic changes, such as obesity, hypertension and other metabolic alterations, including variations in glucose metabolism, while cardiovascular morbidity and metabolic syndrome are among the most important causes of mortality in this patient population [70].

Even though the pathophysiology of BD remains still unclear, substantial evidence from diverse types of studies has currently converged and implicated modifications in neuroplasticity, neuronal connectivity, apoptotic regulation, cell survival and resistance as key mechanisms in the neuropathogenesis of BD [71]. The balance between neuronal cell survival and death is modulated or influenced by several mediators. Changes in the levels of these factors in the periphery of the human body have been proposed as possible biomarkers of BD. The replication of abnormalities in neurotrophins, inflammatory interleukins mediators, monoamine metabolites and oxidative stress species in independent studies leads to the postulation that this association could be regarded as a feasible biomarker and an indicator of BD progression [72].

Regarding the role of neurotrophins in the pathogenesis of BD, it has been elucidated that they belong to a family of proteins associated with neuronal growth, survival, and function [40,73]. BDFN is a mediator of neuronal differentiation and synaptic neuroplasticity. In fact, in the clinical recovery phase, blood serum levels of BDFN are increased, while in the symptomatic phase of the disorder, they are reduced [73]. Polymorphism of the BDNF gene is positively correlated to a higher risk of BD development [74]. In addition, medication, including atypical antipsychotic drugs and lithium can result in an increase in BDFN levels. Notably, side effects due to medication, such as constipation, dizziness, nausea, sexual disorders and memory deficits, contribute to the possible low adherence of some patients to treatment [75]. 

### 4.4. Curcumin in Bipolar Disorder Treatment 

In the last decades, there has been a gradually increasing trend towards traditional medicine and phytochemicals for the treatment of chronic diseases [75]. The effect of curcumin on the outcome of mood swings in people with BD, although not widely studied in humans, may exhibit positive outcomes [68,76]. The international literature has demonstrated curcumin’s involvement in BD molecular pathways. More to the point, curcumin, has shown an ability to interact with diverse biomolecules and increase BDNF levels, also reducing interleukin levels and oxidative stress in people with BD [76]. The BDNF signaling pathway in the hippocampus has been involved in the mechanism of neurogenesis and neuroprotection that preserves homeostasis and provides neuronal survival in humans [77]. The hippocampus is also suggested as a key target for the anti-stress effect of curcumin in both rodents and humans [12,78]. Remarkably, there is strong evidence that curcumin is a major modulator of pro-inflammatory enzymes such as lipoxygenases and cyclooxygenases (COX) as well as certain transcription factors [76]. Curcumin is involved in the signal transmission of inflammatory mediators like IL-6 and IL-1b and it is not only capable of suppressing COX-2 but also further regulates COX-2 gene expression [79]. Subsequently, curcumin can reduce the production of prostanoids in a variety of cell types including macrophages, gastric epithelial cells, and malignant cells [79]. Moreover, curcumin can increase the expression of COX-2, prostaglandin E synthase-1 (mPGES-1) and prostaglandin-I2 synthase (PGI2S) gene expression, which are functionally active [79].

However, a recent randomized control trial on humans with BD has revealed controversial results. Particularly, when nano-formulated curcumin was administrated in combination with different doses of sodium valproate to patients with BD in the maniac phase, no significant differences were observed between the interventional and the control group [53]. In this study, the enrolled patients in both groups received sodium valproate starting at a dose of 600 mg daily and administered up to 20 mg per Kg daily or the highest dosage of the patient’s tolerance [53]. Another study demonstrated significant improvements in depressive symptoms and anti-anxiety effects after curcumin and saffron supplementation [52].

### 4.5. Curcumin and Anxiety and Stress Disorders

Anxiety and stress disorders, which affect almost 10% of the European population and 8% of people worldwide, were the most disabling mental disorders in 2019, demonstrating higher rates during the COVID-19 Pandemic [80]. Depression usually follows or coexists with anxiety and stress disorders [81]. Regarding their pathophysiology, HPA axis function abnormalities and cytokines seem to be involved in stress and anxiety pathogenesis.

Several studies examining curcumin’s efficacy in stress reduction have suggested potential benefits due to its anti-inflammatory, antioxidant, and neuroprotective properties, which could indirectly contribute to stress and anxiety alleviation [8,52,82]. Moreover, when curcumin was combined with fenugreek dietary fiber and administrated orally to people suffering from occupational stress, it exerted significantly positive effects on participants’ mood profiles regarding anxiety and depression [62]. In addition, Gulf War Illness remains a continuing health issue, characterized mostly by anxiety, chronic musculoskeletal pain, depression, fatigue, mood changes and cognitive deficits [83]. In this aspect, curcumin was also tested in treating the severity of symptoms of pain as well as mental disorders in veterans who took part in the Gulf War with promising positive results. Oral administration of 1000 mg/g per day and 4000 mg/g per day reduced disease symptoms’ severity significantly more than placebo at both the lower and higher dosages [61].

## 5. Discussion

The implementation of curcumin in health issues has a history of more than two thousand years ago [2,3,4,5,6,7,8]. Several biological benefits of this turmeric extract have attracted the interest of the scientific community worldwide. Substantial research has demonstrated the properties of curcumin to regulate the inflammation process, to provide wound healing, suppression of advanced glycation end product formation, anticancer activity, protect against neurodegenerative diseases and modulation or treatment of mental disorders [84,85]. In this aspect, this review highlights the effect of curcumin on mental disorders (especially stress and anxiety, depression, major depression and bipolar disorder) in humans by overcoming bioavailability disadvantages.

A plethora of studies have been conducted in vitro or in vivo in animals, while few studies have been conducted in humans. Emerging data suggest curcumin’s anti-depressive properties in humans. Existing randomized control studies and clinical trials investigating the effect of turmeric or curcumin supplementation on depression, major depression, bipolar disorder, anxiety and stress disorders in humans remain still limited. Moreover, the results of these existing clinical studies seem contradictory and inconclusive. In fact, some clinical studies evaluating the effect of curcumin on depression or major depression showed promising results as curcumin improved the mood state of the study participants. In contrast, no differences were found in other surveys. Conversely, other studies found no differences or found some differences in specific participants’ mood conditions. For example, when 1 g of curcumin was administered to obese women, changes in the anxiety scale were observed, whereas no changes were noted in the depression scale compared to the control group [49]. On the other hand, differences were found in the improvement of anxiety and depression (but not stress) when 80 mg of nano-packaged curcumin was administered to 80 diabetic adults [57]. Supplementing 2 g of curcumin per day at the same time as taking antidepressant medication in people aged 31 to 59 years with depression had a positive anti-depressive effect and even a modified effect by reducing inflammatory markers such as IL-1, salivary cortisol levels, TNF-α and BDNF [46].

Notably, in individuals with BD, curcumin in the phase of mania showed no differences, while its effect on the same participants in the depression phase was not examined. Both depression and BD have been associated with vitamin B12 and folic acid deficiencies [86]. The possible cause has been ascribed to the potential polymorphism of the methylenetetrahydrofolate (MTHFR) gene on the total homocysteine (tHcy), folate and B12 levels in patients with BD [86]. In this aspect, the currently available studies did not assess homocysteine, folate and vitamin B12 levels in order to investigate whether participants receiving vitamin B12 and folate supplementation could present better outcomes. Moreover, both anti-anxiety and anti-stress properties of that turmeric compound have also been investigated in humans with contradictory outcomes among the existing studies. Curcumin has also been tested in workers with occupational stress and mild depression. Their outcomes were significant, demonstrating reductions in anxiety, depression and stress scores [45,62]. More to the point, Pandaran Sudheeran et al. explored the safety, antioxidant effectiveness and bioavailability of CurQfen (curcumagalactomannoside (CGM)), a food-grade formulation of natural curcumin with fenugreek dietary fiber that has been shown to possess improved BBB permeability and tissue distribution in rats [62]. In this double-blinded clinical trial, 60 individuals experiencing occupational stress-related anxiety and fatigue were randomly assigned to receive CGM, standard curcumin and placebo for 30 days (500 mg twice daily). The study showed the safety, tolerance and increased efficacy of CGM in comparison with unformulated standard curcumin. Interestingly, a substantial improvement in the quality of life with a considerable decrease in stress, anxiety and fatigue was noted among CGM-treated individuals compared with the standard curcumin group [62]. In support of the above, a cross-sectional study with an 8-week duration evaluated curcumin supplementation and sunlight exposure among office workers with mild depression [45]. This study showed that curcumin supplementation and sunlight exposure programs led to considerable reductions in depression scores and some of its related biomarkers. Such a program could be a sustainable and cost-effective method to attenuate psychological depression among the working class [45].

Despite the extensive and confirmed potency of curcumin, its involvement in signaling pathways and the scientifically confirmed data of its molecular mechanisms of action, the compound presents low bioavailability when administrated orally. Pharmaceutical biotechnology has provided solutions to increase the oral bioavailability of curcumin [87]. Studies on the kinetics and metabolism of curcumin have shown significant results in increasing its oral bioavailability such as the combination with piperine that can interfere with glucuronidation, the use of liposomal curcumin, curcumin nanoparticles, the use of curcumin phospholipid complex, the use of structural analogs of curcumin and the combination with galactomanossides (Fenugreek dietary fiber) with enhanced absorption and half-life in plasma [25].

Moreover, the medicinal and pharmacological properties of the curcumin-C3 complex, which is a compound of three curcuminoids (curcumin, dimethoxy–curcumin and bis–dimethoxy–curcumin) and encapsulated chitosan nanoparticles, were investigated in vitro with positive results regarding the antioxidant and antimicrobial activity of the complex demonstrating over 90% enhanced entrapment efficiency and drug release [84]. It is worth mentioning that Theracurmin, which is curcumin in colloidal submicroscopic particles, is another bioavailable formulation, exhibiting higher oral bioavailability than curcumin. The mean particle size of Theracurmin has been found significantly smaller (0.19 μm) than that of the standard curcumin formulation (22.75 μm) and dispersed consistently [88]. Theracurmin has been tested in cancer patients and patients having cognitive impairment, demonstrating significant anti-neurodegenerative properties and exerting apoptotic effects on human cancer cells, respectively [89,90]. Thus, Theracurmin, a form of colloidal submicroscopic particles, may be considered a more effective bioavailable formulation, which should be examined in future clinical human studies.

Anxiety and stress, particularly chronic stress disorders, are usually treated under the umbrella of depression and not as separate pathological entities. Several randomized control trials have been conducted in humans with depression or BD, investigating the anti-depressant effects of curcumin. Inconsistency is usually attributed to low bioavailability and rapid metabolism of the substance, as already mentioned above. However, when curcumin was administered in a graduated dose or in the form of solid lipid or nanoparticles or a water extract of *Curcuma longa* in healthy humans or patients, the results were found encouraging, as its anxiolytic, anti-depressive and anti-stress activity was supported [48,51,55,56,58,59,60,63,91,92]. Regarding the anti-anxiety and anti-stress effect of *Curcuma longa* on humans, there is a noticeable research gap as most of the studies have been conducted in rodents. It should be noted that in animal studies under induced post-traumatic stress or stress, curcumin was effective in reducing the feeling of fear and anxiety. Of course, animals under conditions of a provoked behavioral disturbance react based on instincts (a typical example is the Pavlovian reflex) [47].

Moreover, the included clinical studies in this review present a great heterogeneity both in terms of the population under study, the screening tools used for mental disorder assessment and the type of mental disorder, as well as curcumin’s optimal dose, the duration of the treatment and the form or combination in which the compound was administrated.

Notably, in the present review, the most significant positive outcomes have been observed when curcumin was administrated in the form of Theracurmin, highlighting greater bioavailability and absorption, providing anti-apoptotic effects and suppression of stress and anxiety levels in cancer patients [48,90] and promoting the stimulus for augmenting the effect of encapsulation of curcumin in nanoparticles on mental disorders and especially on mental disturbances such as anxiety and stress.

During the publishing process of the present review, some novel studies were published concerning curcumin and its bioavailability issues. In a combination of Caco-2 cell monolayer permeability assay, curcumin and piperine combination showed low permeability of curcumin in vitro as compared to a reference item, the dried and crushed turmeric rhizomes [93]. Another recent study aimed to enhance the stability and bioavailability of curcumin using nano-emulsion coating technology [94]. The nano-emulsion system was developed by encapsulating Cur with quaternized chitosan (QMNE) and the nano-emulsion containing curcumin and medium-chain triglyceride (MCT) oil (MNE) was used as a control sample. QMNE demonstrated superior stability, in vitro gastric fluid stability and bioavailability compared to MNE. Moreover, QMNE exhibited excellent emulsification activity and stability. Hence, this recent study has provided valuable insights into the formulation of a system to encapsulate curcumin and the improvement of its stability and bioavailability [94]. Another recent research study constructed composite nanoparticles using abietic acid as a carrier for significantly enhancing the bioavailability of curcumin [95]. In cellular assays, curcumin-loaded abietic acid nanoparticles with the same curcumin concentration were shown to exhibit greater bioaccessibility and bioavailability than free curcumin [95]. Moreover, a green single-step simultaneous aerogel formation-curcumin deposition method was applied to impregnate curcumin into the aerogels through supercritical carbon dioxide (SC-CO_2_) drying technology. The loading of curcumin in aerogels also significantly enhanced the bioaccessibility of curcumin in the simulated gastrointestinal fluid by almost 30-fold when compared to the unloaded curcumin [96]. Based on the current knowledge, the encapsulation of curcumin into nanoparticles seems to be the most promising formulation to effectively increase its bioaccessibility and bioavailability. Thus, there is a strong demand to direct future research in this type of curcumin formulation by performing human clinical studies.

## 6. Conclusions

Curcumin is a natural substance with multifunctional pharmacological properties, which interferes with various neurotransmitter systems and intracellular signaling pathways implicated in mood modulation. Also, curcumin has anti-inflammatory, antioxidant, and neurotrophic effects, suggesting a strong dynamic potential for the treatment of conditions associated with mental disorders pathogenesis. Minimal side effects and low cost make this yellow compound attractive for the therapeutic management of the mentally ill population. Further large-scale clinical studies in humans experiencing depression, major depression, anxiety and stress disorders are strongly recommended to determine the usual dose per mental disorder and the most functional formulation enhancing its clinical utility. Especially, future clinical studies should be focused their attention on the efficiency of the different formulations, and especially of the nanoformulation forms of curcumin or Theracurmin against mental disorders in humans. The encapsulation of curcumin into nanoparticles appears to be the most promising formulation to effectively increase its bioaccessibility and bioavailability, highlighting the strong demand to direct the future research in this type of curcumin formulation by performing human clinical studies.

## Figures and Tables

**Table 1:** Clinical studies in mental disorders and curcumin effectiveness.

Study Type, Participants (N)	Type of Mental Disorder and Screening Tools	Supplementation	Main Results	Ref.
Cross-sectional study, 68 office workers, 2 groups (OG and CG)	Mild depression. At Day 0, Day 30 and Day 60, all subjects were compared in terms of vitamin D, BDNF, IL-6 and PHQ-9.	Curcumin caps: 1000 mg × 2 in the OG. Mindfulness meditation sunlight exposure in both groups, 8 weeks observation.	Vitamin D in Day 0, 30 and Day 30, 60 intervals were significantly higher in OG than in CG. IL-6 serum levels decreased on Day 30 in OG than in CG. Depression scores were lower in OG than in CG at Day 30 and Day 60.	[45]
Randomized, double-blind, placebo-controlled study, 108 male adults	Major Depressive Disorder, The Chinese version of 17-item HDRS, MADRS	1000 mg × 2 curcumin (capsules) or placebo soybean powder daily for 6 weeks on the basis of their current antidepressant medications.	Significant antidepressant behavioral response in depressed patients. ↓ Inflammatory cytokines interleukin 1β and tumor necrosis factor α level and salivary cortisol concentrations. ↑ Plasma BDNF levels. Reports of mild nausea in the curcumin and escitalopram group.	[46]
Randomized, double-blind, placebo-controlled, 2 × 2 factorial design, 152 overweight or obese non-depressed adults (50–80 years) trial	Mental health and Well-Being, POMS questionnaire	Curcumin (160 mg/day), fish oil (2000 mg docosahexaenoic acid + 400 mg eicosapentaenoic acid/day), or a combination of both for 16 weeks.	↓ SMCs (compared to no curcumin treatment). Combining curcumin with fish oil did not result in additive effects.	[47]
Randomized, double-blind, placebo-controlled trial, 60 healthy older adults (60–85 years)	Mood disorders: psychological stress, fatigue, Trait scale of the STAI, BDI-II, Depression, Anxiety and Stress Scales. Chalder Fatigue Scale	A solid lipid curcumin formulation (400 mg as Longvida^®^) was administrated orally (1 h and 3 h after a single dose), chronic (4 weeks) and acute-on-chronic (1 h and 3 h after single dose following chronic treatment.	Significant improvements in fatigue, anxiety and stress compared to control group were observed. No side effects. Hepatocellular safety was retained. LDL levels were reduced.	[48]
RCT, double-blind, cross-over trial, 30 obese patients	Anxiety and depression, BDI-II	Curcumin (1 g/day) or placebo for a period of 30 days. Following a wash-out interval of 2 weeks, each subject was crossed over to the alternative regimen for a further 30 days.	↓ Anxiety symptoms. No significant impact on BDI-II scores.	[49]
Randomized, double-blind, placebo-controlled trial, 56 adults (18–65 y) diagnosed with major depressive disorder	Major depression, IDS-SR30, STAI, various urinary and plasma biomarkers.	2 × 500 mg/day curcumin extract or placebo for 8 weeks.	↑ Urinary thromboxane B2 and substance P. ↓ IDS-SR30 score.	[50]
Randomized double-blind placebo-controlled study, 65 patients (18–63 y)	Major depression, MADRS (1979) and HAM-A	Curcumin (increasing dose from 500 to 1500 mg/day) or placebo for 12 weeks.	Improved MADRS scores. The effects were more pronounced in males compared to females. No statistically significant treatment-emerging adverse effects and no significant effects of curcumin on blood chemistry and ECG measurements. Significant antidepressant effects in participants with MDD. Curcumin administration was safe and well tolerated even when combined with antidepressants.	[51]
Randomized, double-blind, placebo-controlled trial, 123 adults with depression, mean age 43 years, 14 males and 109 females	Major depression, IDS-SR30, STAI	2 × 250 mg/day curcumin extract or 2 × 500 mg/day curcumin extract or 2 × 250 mg/day curcumin extract +15 mg safran extract or placebo for 12 weeks.	Curcumin treatment group associated with significantly greater decrease in IDS-SR30, STAI-state and STAI-trait scores. No differences in efficacy between the active treatment groups. Adverse effects: diarrhea and loose bowels in the high-dose (2 × 500 mg/day) curcumin group.	[52]
Randomized double-blind clinical trial, 55 adults with BD	Bipolar disorder in the mania phase, YMRS, MMSE, CGI	40 mg nano-curcumin (Sinacurcumin) daily, combined with valproic acid medication (600 mg/day + 20 mg/kg) for 4 weeks.	No significant differences between groups.	[53]
Randomized, single-blind trial, 60 adults with MDD	Major depressive disorder, HDRS, CGI	Fluoxetine (20 mg) taken in the morning, or 2 × 500 mg/day curcumin extract or fluoxetine (2 × 20 mg/day) taken in the morning + 2 × 500 mg/day curcumin extract for 6 weeks.	No significance in group differences. Increased frequency of gastritis in the fluoxetine and curcumin group was observed.	[5]
Randomized double-blind control trial, 40 adults 20–40 years old	Depression, CGI-Severity Scale, HDRS, MADRS	500 mg curcumin + 120 mg of ellagic acid (70% concentrate) extracted from pomegranate’s peel, and 50 mg of piperine or placebo together with antidepressants (escitalopram or venlafaxine) 5 weeks	No adverse effects. No significant differences between the groups. Patients in the curcumin group tended to have more rapid relief of depressive symptoms when compared to those in the placebo group.	[54]
Clinical trial, 111 adults with MDD at the age of 18–65 y	Major depressive disorder, HADS, BDI–II	1000 mg/day curcumin extract (C3 Complex) + 10 mg piperine or placebo on standard antidepressant treatment for 6 weeks	↓ HADS anxiety and depression subscale scores and BDI–II affective, somatic and cognitive subscale scores	[55]
Randomized, double-blind, placebo-controlled trial, 70 women with premenstrual syndrome	PMS mental symptoms, PMS symptom self-report questionnaire, serum BDNF measurement.	100 mg × 2 /day curcumin or placebo for 3 consecutive menstrual cycles. Each cycle lasted for 10 days.	Curcumin group showed improvement in mood, physical and behavioral PMS symptoms. ↑ Serum BDNF after 3 months of treatment. No side effects were reported.	[56]
Randomized, double-blind, placebo-controlled trial, 80 adults with type 2 diabetes	Depression and anxiety, DASS-21	80 mg/day nano-curcumin capsules or placebo for 8 weeks.	↓ DASS-21 depression and anxiety score. No reduction in the stress score.	[57]
Single-arm trial, 36 young female with a body mass index ≥ 23 kg/m^2^	Mental health, DASS-21	2 g of turmeric in capsules/day for 90 days.	↓ DASS-21 anxiety score. ↓ Depression or stress scores. No adverse effects were reported.	[58]
Randomized, double-blind, placebo-controlled trial, 79 adults with self-reported digestive complaints (18–65 y)	Depression, anxiety and Stress, DASS-21, SF-36	2 × 500 mg/day curcumin extract or placebo for 8 weeks.	↓ DASS-21 anxiety score and GSRS score. No other between-group differences in other DASS-21 or SF-36 subscale scores. No adverse effects were reported.	[59]
Placebo-controlled trial (randomization details not included), 30 adults (5 males and 25 females) with pulmonary hypertension (18–78 y)	Anxiety and depression, SAS, SDS	60 mg/day of curcumin + conventional antidepressant treatment with the curcumin dose, gradually increasing to 120 mg/day or conventional antidepressant treatment for 3 months.	SAS and SDS scores were significantly lower in the curcumin group compared to control one. No adverse effects were reported.	[60]
Randomized control cross-over trial, 20 men 37–65 years old without current post-traumatic stress disorder or major depression	Gulf War Illness, HADS for screening depression and anxiety severity, Qualtrics Research Suite Offline Application (Qualtrics, Provo, UT, USA) and a Visual Analog Scale for measuring symptoms (pain, fatigue, gastrointestinal distress, cognitive dysfunction, depressed mood, dermatologic complaints and respiratory problems)	Pure Encapsulation’s Curcuma <<Meriva>> turmeric phytosome 100 mg/day (low dose and 4000 mg/day (high dose). Pure Encapsulation’s Boswellia product: 400 mg/day and 800 mg/day. Pure Encapsulation’s Pycnogenol (pine bark extract): 200 mg/day and 400 mg/day, Duration 1 month (30 ± 3 days) of placebo, 1 month (30 ± 3 days) of lower-dose botanical, and 1 month (30 ± 3 days) of higher-dose of each botanical.	Curcumin reduced GWI symptom severity significantly more than placebo at both the lower and higher dose. Maritime pine was more effective than placebo at the higher dose.	[61]
A double-blinded pilot-controlled study, 60 healthy adults working at responsible positions 33 years old 3 groups	Occupational stress, SF-36 Perceived Stress Scale Beck Anxiety Inventory, and SF-36 health survey scales were employed in the study	Curcumin (Curcuma-galactomannoside (500 mg × 2/day, standard *B. curcumin* (500 mg × 2/day) Placebo.	Curcumagalactomannoside group: Significant improvements in quality-of-life score. Significant reduction in stress score. Reduced anxiety, oxidative markers and perceived stress.	[62]
Randomized double-blind placebo-controlled parallel group trial, 48 healthy adults, 20–64 years old 3 groups	Emotional fatigue, anxiety, POMS	Water extract of *C. longa* (WEC): 150 mg WEC and 0.40 mg bisacurone (Low-WEC group) 900 mg WEC and 2.40 mg bisacurone (High-WEC group), Matching placebo tablets (placebo group) daily 8 weeks	POMS score of Low WEC group was significantly lower than the placebo group.	[63]

Observational group, CG: control group, BDNF: brain-derived neurotrophic factor, IL: Interleukin, PHQ-9: patient health questionnaire-9, HDRS: Hamilton depression rating scale, MADRS: Montgomery–Åsberg depression rating scale, POMS: profile of mood states, SMCs: smooth muscle cells, BAI: Beck Anxiety Inventory, BDI-II: Beck Depression Inventory-II: STAI: State-Trait Anxiety Inventory, LDL: low-density lipoprotein, IDS-SR30: Self-rated Inventory of Depressive Symptomatology 30, HAM-A: Hamilton anxiety rating scale, MDD: major depressive disorder, YMRS: young mania rating scale MMSE: mini-mental state examination, CGI: clinical global impression, HADS: hospital anxiety and depression scale, PMS: premenstrual syndrome, DASS: depression anxiety stress scales, SF-36: 36-item short-form survey, SAS: sedation–agitation scale, SDS: self-rating depression scale, WEC: water extract of *C. longa*. ↓ decrease, ↑ increase.

## Data Availability

The data of the present study are available upon request to the corresponding author due to private policy.
